# Editorial: Current trends in the crosstalk between nervous systems and other body systems

**DOI:** 10.3389/fnmol.2023.1157672

**Published:** 2023-02-21

**Authors:** Juan Li, Hongbing Xiang, Jun Xiong

**Affiliations:** ^1^Department of Anesthesiology, Hubei Key Laboratory of Geriatric Anesthesia and Perioperative Brain Health, and Wuhan Clinical Research Center for Geriatric Anesthesia, Tongji Hospital, Tongji Medical College, Huazhong University of Science and Technology, Wuhan, China; ^2^Center for Liver Transplantation, Union Hospital, Tongji Medical College, Huazhong University of Science and Technology, Wuhan, China

**Keywords:** nervous systems, body systems, crosstalk, anesthesia, developing brain, neuroplasticity

The crosstalk between nervous systems and other body systems is complex and is not limited to the communication for neural pathways only. The key role of the nervous system in the control of many involuntary reflexes and reactions to other body systems is generally accepted. Due to the emergence of radiologically targeted neuroimaging technology and neurotropic virus-based transneuronal tracers in recent years, the access to neural connectivity in the other body systems has greatly been improved (Fan et al., 2020; Feng et al., 2020; Huang et al., 2022a). A better understanding of the molecular mechanisms underlying the crosstalk between nervous systems and other body systems may have important implications for the treatment of body injury, age-associated decline, and diseases. This Research Topic in Frontiers in Molecular Neuroscience focuses on current trends in the crosstalk between nervous systems and other body systems.

We first introduce 3 papers that stand their research on anesthesia and brain. Over the two decade there has been a renaissance in our understanding of anesthesia and developing brain. Many studies in rodents have examined the general anesthetics-induced neurotoxicity for developing brains; however, in human, this phenomenon is not as outrageous as it seems. Niu et al. have summarized recent progress made in clinical and preclinical studies to provide useful suggestions and potential therapeutic targets for the protection of the developing brain, and point out that the mechanism of anesthesia-induced neurotoxicity still requires further elucidation in non-human primates. Ma et al. addressed that the role of epigenetic modifications in neurotoxicity induced by neonatal general anesthesia and analyzed the wide prospects of epigenetics in this field and its possibility to work as treatment target. Accumulating evidence show that new methods are beginning to provide key insights into metabolomics and neural circuits of the aged brain. Using the analysis of lipidomics, Mao et al. demonstrated that sevoflurane caused slight changes in lipid metabolism both in the aged brain of marmosets and mice; however, the pathways of lipid metabolism were not affected, suggesting that the effects of sevoflurane on lipid metabolism in aged brains may differ among species.

Between rich functionality and the rather invariant structural architecture of brain neuronal circuits remains one of the major mysteries in neuroscience. Recent progress in understanding the mechanisms of brain-other body systems crosstalk pinpoints the role of the adaptive neuroplasticity. The high incidence of patients with neural circuit-related diseases such as mental disorders, highlights the importance of fundamental research, among others with interventions stimulating adaptive neuroplasticity. Trans synaptic viral tract-tracing in the toolbox of neural labeling methods has been a significant development in the skeletal muscle-brain crosstalk (Huang et al., 2022b). A study by Liu et al. aimed to identify that the basolateral amygdala mediated central mechanosensory feedback of musculoskeletal system. Abdulghani et al. reported the role of the myokine irisin in the muscle-brain crosstalk and reviewed how irisin, which is released in the periphery as well as derived from brain cells, may interact with the mechanisms of cellular autophagy to provide protein recycling and regulation of brain-derived neurotrophic factor (BDNF) signaling *via* glia-mediated control of BDNF maturation, and, therefore, support adaptive neuroplasticity.

The above manuscripts discuss a number of “hot topics” in the crosstalk between nervous systems and other body systems (Figure 1), including anesthetics-induced brain neurotoxicity, skeletal muscle-brain crosstalk, and adaptive neuroplasticity for healthy brain development and maintenance. We hope that studies addressing these topics will inspire other researchers to unravel the unravel the mechanism for the crosstalk between nervous systems and other body systems and promote the translation in clinical neuroplasticity for the treatment of diseases.

**Figure 1:**
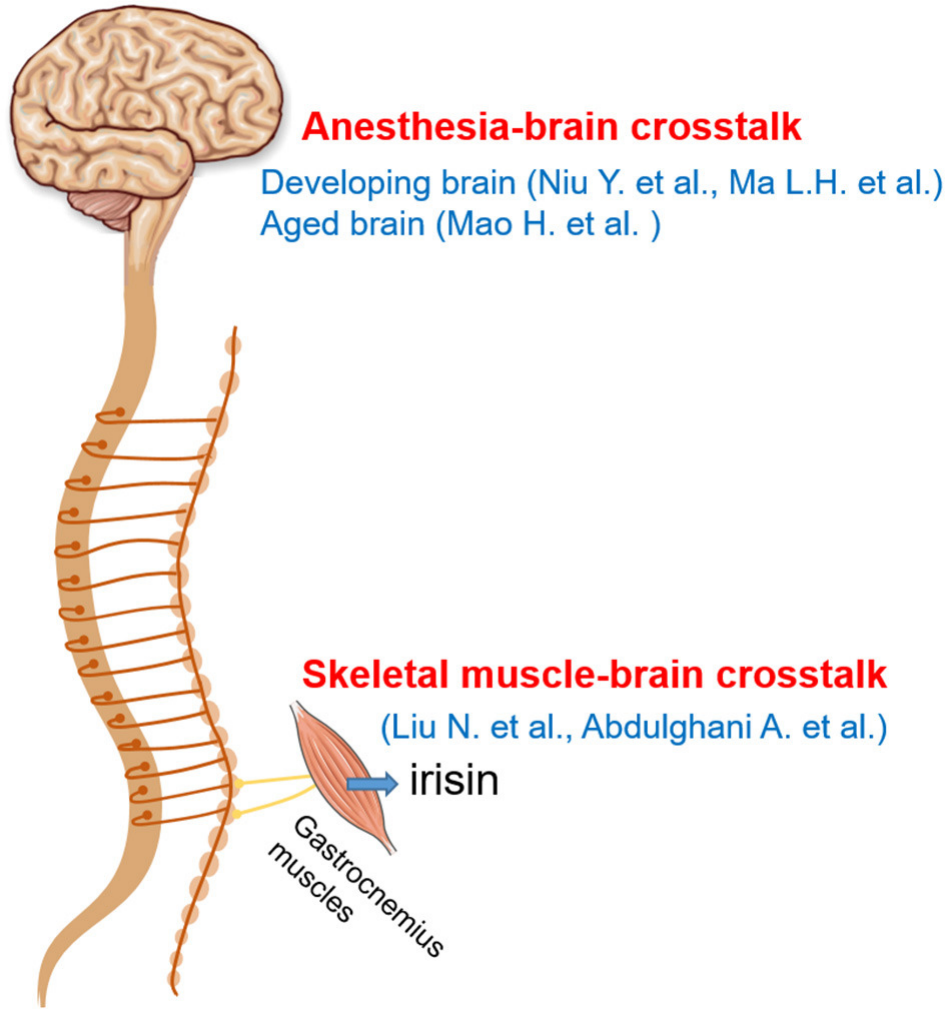
Schematic drawing showing that a number of “hot topics” in the crosstalk between nervous systems and other body, including anesthesia-brain crosstalk (Niu et al.; Ma et al.; Mao et al.) and skeletal muscle-brain crosstalk (Liu et al.; Abdulghani et al.). Some drawings were taken from Fan et al. (2020, Journal of Neuroscience Methods).

## Author contributions

All authors listed have made a substantial, direct, and intellectual contribution to the work and approved it for publication.
